# The energy margin strategy for reducing dose variation due to setup uncertainty in intensity modulated proton therapy (IMPT) delivered with distal edge tracking (DET)

**DOI:** 10.1120/jacmp.v13i5.3863

**Published:** 2012-09-06

**Authors:** Miao Zhang, Ryan T. Flynn, Xiaohu Mo, Thomas Rock Mackie

**Affiliations:** ^1^ Department of Radiation Oncology The Cancer Institute of New Jersey UMDNJ‐Robert Wood Johnson Medical School New Brunswick NJ; ^2^ Radiation Oncology Department University of Iowa Iowa City IA; ^3^ Department of Medical Physics University of Wisconsin‐Madison Madison WI; ^4^ Department of Human Oncology and the Morgridge Institute for Research University of Wisconsin‐Madison Madison WI USA

**Keywords:** EM, intensity‐modulated proton therapy, distal edge tracking, Monte Carlo

## Abstract

Intensity‐modulated proton therapy (IMPT) can produce plans with similar target dose conformity but lower normal tissue dose than intensity‐modulated X‐ray therapy (IMXT). However, due to the finite range of proton beams in tissue, proton therapy treatment plans are usually more sensitive to setup uncertainties than X‐ray therapy plans. In this work, the energy margin (EM) concept, which was initially developed for passive scattering proton therapy, was generalized to apply to IMPT treatment planning. The effectiveness of the EM method was evaluated on five head‐and‐neck cancer patients with distal edge tracking (DET) treatment plans by comparing the original plans (ORG) without an EM to those with an EM. Three beam arrangements were considered: 24 beams delivered over a 360° arc, 12 beams delivered over a 180° arc, and 12 beams delivered over two 90° fan angles. Setup uncertainty was modeled by sampling rigid translational shifts from a Gaussian distribution with a mean of 0 mm and standard deviation of 2 mm in all directions. Delivered dose distributions for all 30 fractions were recalculated using the Geant4 Monte Carlo code. Normalized total dose (NTD) for both the CTV and a ring structure surrounding the PTV were recorded. The plan quality comparison revealed that EM plans had the same CTV coverage but higher dose to the normal tissue than ORG plans. After the simulated delivery, ORG plans resulted in more than 3% underdosage to 5% of the CTV volume in all three beam arrangements, whereas the EM plans did not. Both ORG and EM plans did not produce more than 5% overdose to $D_{2\%}$ of the ring structure. The use of an EM for IMPT treatment planning can substantially reduce sensitivity of the resulting dose distributions to setup uncertainty.

PACS number: 87.55.K‐

## I. INTRODUCTION

Intensity‐modulated proton therapy (IMPT) can produce treatment plans with comparable tumor dose conformity to intensity‐modulated X‐ray therapy (IMXT) with a significantly lower normal tissue integral dose, often by a factor of 2–3, and better critical structure sparing.^1^, ^8^ Two methods for implementing IMPT are three‐dimensional (3D) modulation^(9)^ and distal edge tracking (DET).^(10)^ 3D modulation is the most widely implemented IMPT method^11^, ^12^ in which a 3D grid of proton beam spots, each with individually controllable intensities, is used to cover the whole target region for each beam angle. The intensities of the pencil beams are determined using IMPT treatment planning software.

DET uses only the spots on the distal edge of the tumor volume from a given beam angle, reducing the number of beam spots, but possibly increasing the number of beam directions required to treat the target volume. However, even with multiple beam angles, DET still uses 3 to 10 times fewer spots than 3D modulation.^5^, ^13^, ^14^ DET is expected to reduce IMPT delivery time relative to that of 3D modulation if the time saved by delivering fewer proton pencil beams exceeds the time spent delivering beams from multiple angles by moving the gantry and/or patient table. For pencil beam scanning proton therapy systems (such as the low‐pulse‐rate dielectric wall accelerator)^(15)^ requiring considerably more time to deliver multiple pencil beams, DET would be expected to have greatest saving on delivery time. However, mechanical challenges, such as the difficulty of rotating a bulky proton gantry, must also be considered when delivering DET, requiring that the number of DET beam angles is kept reasonably low.

The finite range of proton beams results a greater sensitivity of IMPT dose distributions to setup and range uncertainties than X‐ray therapy dose distributions. Lomax^(16)^ found both 3D modulation and DET plans had dose variation after the delivery with setup uncertainty. With the same amount of setup uncertainty, DET plans had 2%–3% more dose reduction to the clinical target volume (CTV) than 3D modulation plans. Therefore, the clinical implementation of IMPT necessitates the development of methods to minimize the setup uncertainty‐introduced dose variations. It has been shown^(16)^ that the setup uncertainty in IMPT delivery would introduce significant dose distribution changes in 3D space which cannot be corrected by simply increasing the geometric margin. Many methods have been proposed to reduce the impact of setup uncertainty on IMPT dose distributions produced by 3D modulation. Meyer et al.^(17)^ and Cabal and Jaekel^(18)^ proposed designing a PTV with anisotropic margins in which the lateral margins account for the geometric uncertainty along that direction, and the margins along the beam axis account for both geometric and range uncertainties. Pflugfelder et al.^(19)^ proposed reducing the intensity of the beam spots that are most sensitive to patient motion based on their location relative to tissue heterogeneities. Several groups of investigators^20^, ^23^ incorporated the uncertainties into the treatment planning process using probabilistic and robust optimization techniques.

The energy margin (EM) technique, originally developed for passive scattering proton therapy by Urie et al.,^(24)^ is a simple method for reducing the sensitivity of proton therapy dose distributions to setup uncertainties. For a given proton beam, the EM method uses a larger aperture size to accommodate lateral target motion and a thinner compensator to accommodate the range uncertainty. The concept of EM has also been used in IMPT but only to a limited extent — for example, by uniformly adding an EM to account for the systematic range uncertainty in the CT number conversion.^(9)^ In the current work, the EM is applied to DET, the most sensitive IMPT delivery case, in order to investigate the possibility of using the EM to reduce the dose variation introduced by setup uncertainties. A new way of designing the EM for each individual proton pencil beam considering the amount and the direction of setup uncertainty is presented, and quantitatively assessed by treatment plan comparisons.

## II. MATERIALS AND METHODS

### A. EM determination

Assume the PTV was contoured partially following the definition in ICRU Report 78,^(25)^ which considered the margin for setup uncertainty but not the internal margin for intrafractional motion or tumor shrinkage. DET‐based IMPT planning^(13)^ consists of defining the beam angles and the spot spacing, determining the nominal pencil beam energies necessary to reach the distal edge of the PTV, calculating the dose distribution for each pencil beam, and optimizing the individual pencil beam intensities and calculating the total dose distribution.

**Figure 1:**
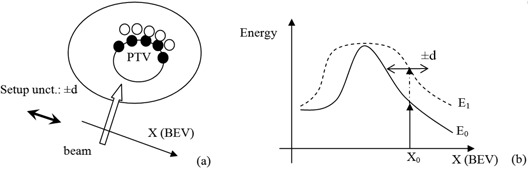
EM determination in DET‐based IMPT considering setup uncertainties. (a): Schematic drawing of beam direction (open arrow), original spot location (black dots) and updated spot location (open circle) of a DET plan. The axis normal to the beam direction is the x‐axis in beam's eye view (BEV). The thick double arrow indicates the direction of translation due to setup uncertainty projected along the x‐axis of the BEV. The magnitude of the setup uncertainty along the X direction is ±d. After adding an EM, the spots' locations move further into the normal tissue. (b): Energy vs. location for each pencil beam along the x‐axis of the BEV. The solid line ($E_{0}$) is the pencil beam energy determined from static image which is represented by the black dots in (a). The dashed line ($E_{1}$) is determined after simulating setup uncertainty which is represented by the open circles in (a).

Patient setup uncertainties can result in under‐ or overshoot of individual pencil beams relative to the distal edge of the PTV, resulting in PTV underdose or normal tissue overdose, respectively. A simple solution to avoid PTV underdose due to beam undershoot is to assign each pencil beam a “safety energy” which is greater than that required to reach the distal edge of the PTV on planning CT. Thus when the range of pencil beam is shortened due to setup uncertainty, it still can reach the distal edge of the PTV. Since setup uncertainty is limited in magnitude, there exists for each individual pencil beam a minimum energy that guarantees that the pencil beam will reach the designated location during the course of treatment. For any pencil beam, that minimum energy would be higher than, or at least equal to, the energy value determined from the static planning CT image. The EM is the difference between the minimum and nominal energies, and can be determined by simulating possible setup variations based on the initial planning CT scan. An unrealistic example would be utilizing a complete set of patient CT images of each treatment day available at the time of planning. Different pencil beam energy values to reach the same single spot could be found from those series of CT images. If the pencil beam was assigned the highest energy among those values, it will always reach the spot during the course of treatment. Hence, that energy is the minimum energy required. In reality, if setup uncertainty is estimated and can be modeled as a rigid patient shift, the minimum energy can be accurately found by using the planning CT image alone. Fig. 1 demonstrates the selection of EM for single pencil beam. The minimum energy required for a given proton pencil beam is determined as follows:

(1) $$E_{1}({\vec{x}}_{0})=\text{max}\{E_{0}(\vec{x}),{\vec{x}}_{0}‐\vec{d}\leq \vec{x}\leq {\vec{x}}_{0}+\vec{d}\}$$

where *x* is the vector in the plane perpendicular to the pencil beam direction, *E(x)* is the nominal energy profile of the distal edge of the target, and *d* is the maximum setup uncertainty projected on the plane perpendicular to the pencil beam direction. Equation (1) is applied in the beam's eye view (BEV). Since any rigid motion along the beam direction would not change the proton range for that pencil beam, the component of the setup uncertainty parallel to the pencil beam direction would not affect the range of the pencil beam, and was not considered. The dashed curve in (Fig. 1b) shows the minimum required energy $E_{1}$ versus the location of the pencil beams.

Equation (1) suggests that an EM‐based DET treatment plan would deliver higher normal tissue doses, since beam spots with the safety energy would be placed outside the PTV. However, this would not be the final result since the treatment plan optimization process reduces normal tissue overdose by penalizing the intensities of those “overshoot” pencil beams. A large EM is most likely associated with pencil beams either close to a heterogeneity boundary or tangential to the patient surface. Those pencil beams, naturally less preferred to be used, will be ‘tagged’ during energy determination by adding a large EM. In the following optimization process, the optimizer will notice those ‘tagged’ pencil beams and selectively lower their weights according to the dose objectives.

### B. Patient selection and treatment planning

**Figure 2:**
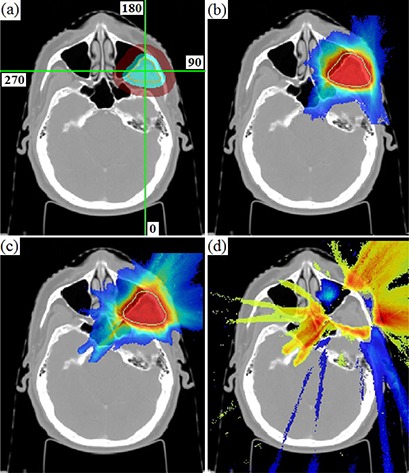
Anatomy of one head and neck patient (a) with the contours of the CTV (orange contour), the PTV (solid blue), and the ring structure (solid red); the bright green cross lines show the center of the CTV. Planned dose distribution of the full arc ORG (b) and the full arc EM (c), and dose difference between those two plans (d). Red/yellow in (d) shows regions received higher dose from the EM than that from the ORG, whereas blue indicates regions received less dose.

In the head and neck region, without any significant internal organ motion, setup uncertainty is the major component of the geometric uncertainty. In the same region, air cavities and uneven patient surfaces pose IMPT delivery challenges. Large heterogeneities enhance the dose variation with even small setup uncertainty, thus is a challenging site to accurately treat with IMPT. Comparison plans with the using of EM (EM plan) and without the using of EM (original (ORG) plan) were generated to test the effectiveness of the EM on five head‐and‐neck cancer patients. In order to ensure a simple comparison, each of the five patients had only one CTV. The PTV was defined as the CTV plus a 5 mm isotropic margin, and was prescribed 60 Gy in 30 fractions of 2 Gy each. A 10 mm ring structure surrounding the PTV was generated in all five cases to represent the worst possible location of adjacent critical organs. Figure 2 shows the anatomy of one of the five patients who had the tumor directly adjacent to the sinus cavity. The maximum dose allowed to the ring and the PTV was 60 Gy. The minimum dose allowed to the PTV was 58 Gy. To minimize the dose spilling out from the PTV, we required that no greater than 3% of the ring volume could receive a dose greater than 54 Gy.

Three DET plans with different beam arrangements — full arc, half arc, and two‐fan — were generated. Arc or fan treatments denote angular arcs over which the proton gantry rotates, but the gantry is not assumed to rotate continuously. This terminology is different from that used for X‐ray arc therapy in which the beam delivery continues while the gantry is rotating. The full arc was composed of 24 uniformly spaced static beam angles over a full 360°. In reality, a full arc would only be used if the tumor were near the center of the patient. Cases such as the one shown in Fig. 2 would employ selected directions that spare beams coming through longer paths of normal tissue. The half arc had 12 uniformly spaced static beam angles over 180°. The two‐fan arrangement was composed of 12 uniformly spaced static beam angles forming two opposing 90° arcs. Locations of the half arc and the two‐fan were customized for each patient to reduce the irradiated volume of normal tissue. For the patient shown in (Fig. 2a), the half‐arc beam extended from 45° to 225°. The two‐fan beam arrangement was composed of two arcs: 0° to 90°, and 180° to 270°. The angles are shown in (Fig. 2a).

All treatment plans were generated using pencil beams with 3 mm lateral Gaussian standard deviations in air at isocenter, and 3 mm lateral spot spacing. The energy spread for each pencil beam was assumed to be a Gaussian distribution centered on the nominal energy, with a 1% standard deviation. The energy resolution was 1 MeV. The simulated setup uncertainty used to determine the energy margin was assumed to be rotation free and within 4 mm for each direction with the maximum shift in 3D of 6 mm (see Section II.C). The motion related parameter in Eq. (1), *d*, was fixed at 6 mm for all the beam directions. Some plan information is summarized in Table 1.

**Table 1:** Plan information of five patients. The spot number is for a full arc plan. The half arc and two‐fan plans had half the number of spots as the full arc plan.

*Patient #*	*PTV Volume (cc)*	*Spot #*	*ORG* $E_{\text{ave}}$ *(MeV)*	${\mathrm{EME}}_{\text{ave}}$ *(MeV)*
1	27.4	3686	103.62	110.87
2	72.7	7989	121.85	130.26
3	59.2	5387	112.87	121.43
4	67.4	7495	110.99	119.58
5	32.1	4366	110.83	120.79

The Geant4.9.1.p1 Monte Carlo code^(26)^ was used for all proton dose calculations.^27^, ^29^ Only protons and neutrons were transported for the dose calculation, and all other secondary particles were assumed to deposit their energy at the location where they were generated. CT images with voxel size of 0.98 mm x 0.98 mm x 2.5 mm was made to represent a patient in Geant4. The material for each voxel was determined by using density range values listed in Table 2. The material composition from the NIST website^(30)^ was used. Dose‐to‐water was calculated by scaling each voxel's dose by the mass stopping power ratio of the voxel's material to that of water. For each proton pencil beam, 10^4^ primary proton histories were simulated. With 10^3^ proton pencil beams typically presented in each plan, the final dose distribution for a plan was composed by transporting on the order of 10^7^ primary proton histories. Pencil beam intensities were optimized using an iterative linear least‐squares method.^(31)^

**Table 2:** Density threshold for Monte Carlo material construction.

*Material*	*Density Range (g/cm* ^*3*^ *)*
Air	<0.207
Adipose tissue	0.207 < ρ < 0.979
Water	0.979 < ρ < 1.0
Muscle	1.0 < ρ < 1.109
Dense bone	>1.109

### C. Setup uncertainty simulation and biological dose summation

The setup shift values for each individual fraction along the anterior–posterior, superior–inferior, and lateral directions were sampled from a Gaussian distribution with mean and standard deviation (a) of 0 mm and 2 mm, respectively, and a cutoff of ±4 mm, which are within the clinical head‐and‐neck cancer setup uncertainties reviewed by Hurkmans et al.^(32)^ For each patient, one set of shift values for 30 fractions was used to simulate the delivery of both the ORG and the EM plans. By setting the mean value of the Gaussian to be 0 mm, we assumed the shift was a random setup error rather than a systematic setup error. The dose distributions were recalculated for each patient shift.

In order to account for the radiobiological effects of fractionation, dosimetric results were expressed in terms of normalized total dose (NTD) as follows:

(2) $$NTD=\sum_{i=1}^{N}d_{i}\times \frac{\left(\alpha /\beta +d_{i}\right)}{\left(\alpha /\beta +d_{ref}\right)}$$

The summation runs through fraction 1 to N, with dose from each fraction denoted as *di*. The generally accepted value^33^, ^34^ of α/β=10 Gy for head and neck cancer (CTV), α/β=1.5 Gy for normal tissue (ring), and 2 Gy of $d_{\text{ref}}$ was used in this study. Since the dose included the effects of setup uncertainty explicitly, the relevant dose was the dose to the CTV rather than the PTV. Dose to the ring structure representing the normal tissue around the PTV was also evaluated as a surrogate for normal structures in the high dose/high dose gradient region.

## III. RESULTS

**Figure 3:**
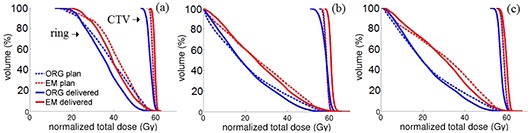
The CTV and the ring DVHs from the patient shown in with three different beam arrangements: (a) full arc, (b) half arc, and (c) two‐fan.

The dose distribution on one slice of the full arc ORG and EM plans from one patient is shown in (Figs. 2b)and (2c). Figure 3 shows the planned and delivered DVHs for the CTV and the ring structure for three different beam arrangements in one patient's case. The ORG and EM plans yielded identical planned CTV DVHs. After 30 fractions of delivery, the CTV DVHs after delivery were lower than those from the ORG plan. For the EM plan, the delivered CTV DVH was greater than or equal to the planned for any volume point. The planned dose to the ring structure was higher in the EM plan than that in the ORG plan.

### A. CTV

**Figure 4:**
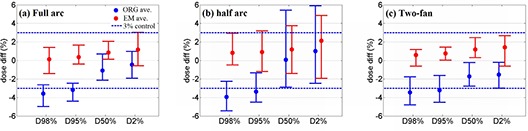
Dose‐volume variation for the CTV. The vertical axis shows dose‐volume variation in terms of percentage to the 60 Gy prescription. The markers and bars represent the mean and the range of the dose‐volume variations over all five patients.

Dose‐volume variation plots are shown in Fig. 4, which show the average and the range (over all five patients) of the planned minus delivered DVH‐values for several volume points. A robust plan should have a dose‐volume variation close to zero; positive and negative dose‐volume variations are preferred for tumor and normal tissue, respectively.

The delivered ORG plans generally underdosed the CTV resulting in negative dose‐volume variation. The CTV dose distributions for the EM plans were more robust to delivery uncertainty than those from the ORG plans. If ±3% of the prescription dose was considered the clinically acceptable threshold of dose variation on every CTV volume point, as the two dashed lines in Fig. 4 show, plans within those lines would be acceptable for delivery. In such a case, only the full arc and the two‐fan EM plans were acceptably robust with respect to setup uncertainty. If we further loosen the threshold by allowing more than 3% overdose but no less than 3% underdose to the CTV, all EM plans would be considered acceptable for delivery. For the ORG plans, the dose‐volume variation on CTV $D_{98\%}$ and $D_{95\%}$ were almost always below the 3% underdose threshold, which would not be considered acceptable for delivery.

### B. Ring structure

**Figure 5:**
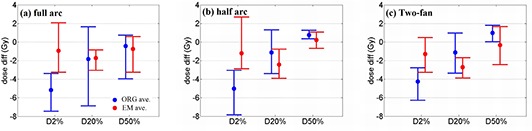
Dose‐volume variation for the ring structure. The markers and bars represent the mean and the range of the dose‐volume variations over all five patients.

Dose‐volume variation to percentage volume of the ring structure ($D_{2\%}$, $D_{20\%}$, and $D_{50\%}$) between the ORG and EM plans are plotted in Fig. 5. In most cases, the average dose‐volume variation for both the ORG and the EM plans was below 0 Gy. This means setup uncertainty had a blurring effect on the planned dose distributions, and the surrounding normal tissue received a lower dose than the planned. As for the high‐dose region on the ring structure, as $D_{2\%}$ represents, the blurring effect was stronger in the ORG plans than that in the EM plans. For the other dose regions as $D_{20\%}$ and $D_{50\%}$ indicates, the dose‐volume variation was similar between the ORG and the EM plans. Since the normal tissue tended to receive lower doses than suggested in the plan in the most cases, regardless of using the treatment planning method, both ORG and the EM were considered comparable at ensuring ring structure dose robustness.

## IV. DISCUSSION

### A. Applying an EM to other types of motion and other types of proton delivery

The EM concept can be generalized to account for other sources of proton range variation during the treatment, such as internal organ motion, if the effects of the motion can be quantified. However, the actual clinical investigation is outside the scope of the current study and will not be discussed here.

**Figure 6:**
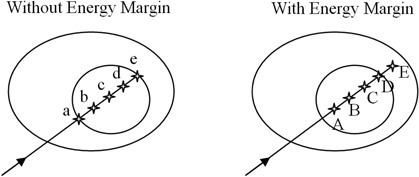
Illustration of EM used in 3D modulation based IMPT. The inner circle is the PTV. The arrow indicates the pencil beam direction. Stars denote locations of spots.

The EM method can be implemented for 3D modulation‐based IMPT using Eq. (1). The resulting IMPT plan may not effectively reduce the dose variation due to setup uncertainty because of the fixed planning work flow; the energy determination is followed by the optimization. The major difference between 3D modulation and DET, in terms of planning, is that 3D modulation uses a different spot grid covering the whole target region. As the left panel of Fig. 6 shows, for any given spot (e.g., spot a), there are always several other spots (b‐e) sharing the same beam path with changing energy in 3D modulation. If we added EMs to pencil beams a‐e, they would reach spots A‐E showing in the right panel of Fig. 6 on the planning CT image. With a prescription of a uniform dose on the PTV and less dose on any other normal tissue, only spots A‐D would be selected by the optimizer, whereas the pencil beam aiming at spot E would be excluded to reduce the normal tissue dose. The analogy of this situation would be having a spot grid covering a region larger than the PTV in the initial beam setup. After going through optimization, only spots within the PTV volume would remain to meet the dose objectives. Therefore, the EM plan's robustness will be no different from the one without EM in the 3D modulation‐based IMPT. To use EM effectively in reducing motion‐introduced dose variation in the 3D modulation‐based IMPT, the implementation has to be modified. However, this investigation is beyond the scope of this study and will not be discussed here.

### B. Comparing EM with other solutions

Two solutions in addition to the EM approach — robust optimization^21^, ^23^ and an anisotropic PTV margin^17^, ^18^ — have been proposed to reduce the geometric and range uncertainty on dose distributions in proton therapy. The anisotropic margin method differentiates the lateral margin, which only accounts for geometric uncertainty, from the longitudinal margin, which includes the proton range uncertainty on top of the geometric uncertainty. Individual beams from different directions would have different PTV definitions. Such an approach is effective if all beams in a plan were also optimized individually, which is the case for current 3D modulation planning.^17^, ^18^ However, when all the beams are required to be optimized at the same time, it is difficult to define the objective functions for multiple PTVs. The robust optimization solution is fundamentally different from the conventional optimization in that the geometric uncertainty distribution would be incorporated directly into the optimization process. In that case, PTV generation is not mandatory in the treatment planning process.^21^, ^23^

The EM solution more closely resembles conventional X‐ray therapy treatment planning: the design of the PTV only considers the internal margin and the setup margin, the dose objectives are specified to the PTV, and formulation of the objective function does not account for robustness. The only extra information required is the limit of the proton range uncertainty to design the EM, making the EM approach simpler.

### C. Additional remarks

The EM improved the robustness of the CTV dose coverage relative to the ORG method at the expense of increased normal tissue dose. The ring structure in this study was a challenging case compared to most clinical situations in which the critical organ is either spatially separated from the PTV or abutting the PTV on one side. By using directional blocked beams (e.g., the two‐fan beam arrangement), the dose‐to‐critical organs, in most cases, could be less than what the ring structure received in this study. However, there are situations even more challenging than the one we simulated (e.g., tumor abutting the spinal cord). In cases like that, having critical organs with tight dose tolerance and the tumor adjacent to each other, tight dose constraints may need to be applied to the critical organ, which in turn may compromise the target dose coverage. To ensure the target coverage and the critical organ sparing, better immobilization and online imaging which can reduce the range uncertainty, and hence the EM, will be more helpful to secure a robust plan delivery.

## V. CONCLUSIONS

EM plans were more robust to the setup uncertainty than ORG plans; however, due to the extra energy used for each pencil beam, normal tissue around the PTV received higher dose in the EM plans than the ORG plans.

## ACKNOWLEDGMENTS

We would like thank Dr. Paganetti from MGH who kindly provided the physics module for Geant4 proton simulation. The authors would also like to thank Dr. Paul Wilson, Dr. Søren Bentzen, and Dr. David Westerly for their helpful discussions on this work.
